# Post-earthquake health-service support, Nepal

**DOI:** 10.2471/BLT.17.205666

**Published:** 2018-02-05

**Authors:** Sophie Goyet, Rajan Rayamajhi, Badry Nath Gyawali, Bhola Ram Shrestha, Guna Raj Lohani, Damodar Adhikari, Edwin Salvador, Roderico Ofrin, Jos Vandelaer, Reuben Samuel

**Affiliations:** a Nepal Country Office, World Health Organization, United Nations House, Pulchowk, Lalitpur, Kathmandu, Post Box Number 108, Nepal.; b Epidemiology and Diseases Control Division, Ministry of Health, Government of Nepal, Kathmandu, Nepal.; c Curative Service Division, Ministry of Health, Government of Nepal, Kathmandu, Nepal.; d Bangladesh Country Office, World Health Organization, Dhaka, Bangladesh.; e South-East Asia Regional Office, World Health Organization, New Delhi, India.

## Abstract

**Problem:**

Seven months after the April 2015 Nepal earthquake, and as relief efforts were scaling down, health authorities faced ongoing challenges in health-service provision and disease surveillance reporting.

**Approach:**

In January 2016, the World Health Organization recruited and trained 12 Nepalese medical doctors to provide technical assistance to the health authorities in the most affected districts by the earthquake. These emergency support officers monitored the recovery of health services and reconstruction of health facilities, monitored stocks of essential medicines, facilitated disease surveillance reporting to the health ministry and assisted in outbreak investigations.

**Local setting:**

In December 2015 the people most affected by the earthquake were still living in temporary shelters, provision of health services was limited and only five out of 14 earthquake-affected districts were reporting surveillance data to the health ministry.

**Relevant changes:**

From mid-2016, health facilities were gradually able to provide the same level of services as in unaffected areas, including paediatric and adolescent services, follow-up of tuberculosis patients, management of respiratory infections and first aid. The number of districts reporting surveillance data to the health ministry increased to 13 out of 14. The proportion of health facilities reporting medicine stock-outs decreased over 2016. Verifying rumours of disease outbreaks with field-level evidence, and early detection and containment of outbreaks, allowed district health authorities to focus on recovery and reconstruction.

**Lessons learnt:**

Local medical doctors with suitable experience and training can augment the disaster recovery efforts of health authorities and alleviate their burden of work in managing public health challenges during the recovery phase.

## Résumé

**Problème:**

Sept mois après le séisme survenu en avril 2015 au Népal, et alors que les opérations de secours s'essoufflaient, les autorités sanitaires ont été confrontées à des difficultés continues dans la prestation de services de soins et la communication en matière de surveillance des maladies.

**Approche:**

En janvier 2016, l'Organisation mondiale de la Santé a recruté et formé 12 médecins népalais afin de fournir une assistance technique aux autorités sanitaires dans les districts les plus affectés par le séisme. Ces intervenants supplémentaires d'urgence ont supervisé la reprise des services de soins et la reconstruction des établissements de soins, ont contrôlé les stocks de médicaments essentiels, ont facilité la communication avec le ministère de la Santé quant à la surveillance des maladies et ont participé aux enquêtes sur les flambées épidémiques.

**Environnement local:**

En décembre 2015, les personnes les plus affectées par le séisme vivaient encore dans des abris temporaires, la prestation de services de soins était limitée et seuls cinq districts affectés par le séisme sur 14 communiquaient des données de surveillance au ministère de la Santé.

**Changements significatifs:**

Depuis le milieu de l'année 2016, les établissements de soins ont progressivement été en mesure d'offrir le même niveau de services que dans les zones non affectées, avec notamment des services pédiatriques et destinés aux adolescents, le suivi des patients atteints de tuberculose, la prise en charge des infections respiratoires et les premiers secours. Le nombre de districts transmettant des données de surveillance au ministère de la Santé est passé à 13 sur 14. Le nombre d'établissements de soins en rupture de stock de médicaments a peu à peu diminué en 2016. La vérification des rumeurs de flambées épidémiques par des preuves recueillies sur le terrain, ainsi que la détection et l'endiguement précoces des épidémies, ont permis aux autorités sanitaires des districts de se concentrer sur la reprise et la reconstruction.

**Leçons tirées:**

L'appui de médecins locaux expérimentés et formés peut seconder les efforts de relèvement après catastrophe des autorités sanitaires et alléger leur charge de travail en matière de gestion des problèmes de santé publique durant la phase de relèvement.

## Resumen

**Situación:**

Siete meses después del terremoto en abril de 2015 en Nepal, a medida que disminuyeron los esfuerzos de ayuda humanitaria, las autoridades de salud tuvieron que enfrentar continuos desafíos para la provisión de servicios de salud y para la notificación de la vigilancia de enfermedades.

**Enfoque:**

En enero de 2016, la Organización Mundial de la Salud reclutó y capacitó a 12 médicos nepaleses para proporcionar asistencia técnica a las autoridades de salud en los distritos más afectados por el terremoto. Estos oficiales de apoyo supervisaron la recuperación de los servicios de salud y la reconstrucción de los establecimientos de salud, supervisaron el suministro de medicamentos esenciales, facilitaron los informes de vigilancia de enfermedades al Ministerio de Salud y asistieron en las investigaciones de brotes.

**Marco regional:**

En diciembre de 2015, las personas más afectadas por el terremoto seguían viviendo en alojamientos temporales, la provisión de servicios de salud era limitada y apenas cinco de los 14 distritos afectados por el terremoto realizaban informes sobre datos de vigilancia al Ministerio de Salud.

**Cambios importantes:**

Desde mediados de 2016, los centros de salud pudieron brindar gradualmente el mismo nivel de servicios que en las áreas no afectadas, incluidos los servicios pediátricos y de adolescentes, el seguimiento de pacientes con tuberculosis, el manejo de infecciones respiratorias y primeros auxilios. El número de distritos que realizaron informes sobre datos de vigilancia al Ministerio de Salud aumentó a 13 de 14. La proporción de establecimientos de salud que informaron escasez de medicamentos disminuyó en 2016. La verificación de rumores sobre brotes de enfermedades con pruebas a nivel de campo, la detección temprana y la contención de los brotes permitió que las autoridades de salud del distrito pudieran centrarse en la recuperación y la reconstrucción.

**Lecciones aprendidas:**

Los médicos locales, con la experiencia y capacitación adecuadas, pueden contribuir a las iniciativas de las autoridades de salud en situaciones de desastre y aliviar su carga de trabajo al gestionar los desafíos de salud pública durante la fase de recuperación.

## ملخص

**المشكلة:**

بعد مرور سبعة أشهر على وقوع زلزال نيبال في شهر أبريل/نيسان 2015، ومع تراجع حجم جهود الإغاثة، فقد واجهت السلطات الصحية تحديات مستمرة تتعلق بتقديم الخدمة الصحية، وتقديم التقارير حول رصد الكارثة.

**الأسلوب:**

عمدت منظمة الصحة العالمية في شهر يناير/كانون الثاني من عام 2016 إلى انتداب 12 طبيبًا نيباليًا وتدريبهم على أعمال تقديم المساعدة المتخصصة للسلطات الصحية في الأقاليم الأكثر تضررًا من الزلزال. وقد تولى هؤلاء المختصون بتقديم الدعم في حالات الطوارئ متابعة عملية استعادة الخدمات الصحية وإعادة تأسيس المرافق الصحية، كما رصدوا مخزون الأدوية الضرورية، وقاموا بتيسير أعمال تقديم تقارير متابعة الأمراض إلى وزارة الصحة، كما ساعدوا في جهود تقصي حالات تفشي الأمراض.

**المواقع المحلية:**

في شهر ديسمبر/كانون الأول من عام 2015، كان الأشخاص الأشد تأثرًا بالزلزال لا يزالون يعيشون في ملاجئ مؤقتة، وكان تقديم الخدمات الصحية محدودًا، واقتصر رفع التقارير الخاصة ببيانات الرصد إلى وزارة الصحة على خمسة أقاليم من بين 14 إقليمًا متأثرًا بالزلزال.

**التغيّرات ذات الصلة:**

بدأت المرافق الصحية في استعادة قدرتها على تقديم الخدمات الصحية بنفس المستوى المقدم في المناطق غير المتأثرة اعتبارًا من أواسط عام 2016، بما يشمل خدمات طب الأطفال والمراهقين، ومتابعة مرضى السل، وإدارة حالات عدوى الجهاز التنفسي، وتقديم الإسعافات الأولية. وتزايد عدد الأقاليم التي تبلغ عن بيانات الرصد إلى وزارة الصحة ليصل إلى 13 إقليمًا من بين 14 إقليمًا. وانخفضت نسبة المرافق الصحية التي تبلغ عن نفاد مخزون الأدوية لديها على مدار عام 2016. وكان التحقق من شائعات تفشي الأمراض من خلال تحري الأدلة على المستوى الميداني والكشف المبكر عن حالات التفشي واحتوائها قد أتاح للسلطات الصحية بالأقاليم التركيز على استعادة الخدمات وإعادة تأسيسها.

**الدروس المستفادة:**

يمكن للأطباء المحليين ذوي الخبرة والتدريب المناسبين استكمال جهود السلطات الصحية الهادفة إلى تجاوز آثار الأزمة، وتخفيف العبء الناشئ عنها في مجال إدارة تحديات الصحة العمومية خلال مرحلة استعادة الخدمات.

## 摘要

**问题:**

距 2015 年 4 月尼泊尔地震发生 7 个月后，随着救援工作的缩减，卫生当局在卫生服务提供和疾病监测报道方面面临着持续挑战。

**方法:**

2016 年 1 月，世界卫生组织招募并培训了 12 名尼泊尔医生，向地震中受灾最严重地区的卫生当局提供技术援助。这些紧急支援官员监督卫生服务的恢复和医疗机构的重建、监控基本药物库存、协助向卫生部报告疾病监测情况并协助疫情调查。

**当地环境:**

在 2015 年 12 月，受地震影响最严重的人们仍然住在临时避难所，提供的卫生服务很有限，14 个受灾地区中只有 5 个向卫生部报告监测数据。

**相关变化:**

从 2016 年年中开始，医疗机构逐渐能够提供与未受影响地区相同的服务水平，包括儿科和青少年服务、肺结核患者随访、呼吸道感染管理和急救。在 14 个受灾地区中，向卫生部报告监测数据的地区数量增加到 13 个。报告药品库存的医疗机构比例比 2016 年有所下降。通过实地证据核实疫情爆发的传闻，及早发现和控制疫情，使地区卫生当局能够专注于恢复和重建工作。

**经验教训:**

具有适当经验、接受过培训的当地医生可增强卫生当局的灾后恢复工作，并减轻其在恢复阶段处理公共卫生挑战的工作负担。

## Резюме

**Проблема:**

Спустя семь месяцев после землетрясения, произошедшего в Непале в апреле 2015 года, по мере сокращения усилий по оказанию помощи органы здравоохранения столкнулись с постоянными проблемами в области предоставления медицинских услуг и отчетности в области эпиднадзора.

**Подход:**

В январе 2016 года Всемирная организация здравоохранения набрала и обучила 12 непальских врачей для оказания технической помощи органам здравоохранения в районах, наиболее пострадавших в результате землетрясения. Эти сотрудники службы экстренной помощи контролировали восстановление службы здравоохранения и реконструкцию медицинских учреждений, проводили мониторинг запасов основных лекарственных средств, оказывали содействие в предоставлении отчетности в области эпиднадзора в Министерство здравоохранения и помогали в расследовании вспышек заболеваний.

**Местные условия:**

В декабре 2015 года наиболее пострадавшие от землетрясения люди все еще жили во временных убежищах, предоставление медицинских услуг было ограниченно, только пять из 14 пострадавших от землетрясения районов предоставляли отчетность о заболеваниях в Министерство здравоохранения.

**Осуществленные перемены:**

С середины 2016 года медицинские учреждения, включая детские медицинские учреждения, службы в области диагностики и лечения туберкулеза, постепенно смогли предоставлять тот же уровень услуг, что и в незатронутых районах, а также восстановили уровень лечения респираторных инфекций и оказания первой медицинской помощи. Количество районов, предоставляющих отчетность о заболеваниях в Министерство здравоохранения, увеличилось до 13 из 14. В 2016 году снизилась доля медицинских учреждений, сообщивших о дефиците запасов лекарственных средств. Проверка слухов о вспышках заболеваний с помощью данных, полученных на местах, а также раннее выявление и сдерживание вспышек заболеваний позволили районным органам здравоохранения сосредоточиться на восстановлении и реконструкции.

**Выводы:**

Местные врачи с соответствующим опытом, прошедшие обучение, могут активизировать усилия по восстановлению после стихийных бедствий органов здравоохранения и облегчить бремя работы по решению проблем общественного здравоохранения на этапе восстановления.

## Introduction

Major disasters can have a severe health impact on populations and health systems, especially in underprepared low- and middle-income countries.^1^ The huge efforts undertaken for the immediate response can often be sustained only for a few weeks. Yet disasters have long-term consequences and their management encompasses more than immediate interventions.^2^ Affected health systems must be restored, actual and potential health risks mitigated, and communities enabled to prepare better for future disasters.^3^

More attention is being given to post-disaster response. Whereas the immediate response to the Nepal earthquake of 2015 has already been well described,^4^^–^^7^ there is a lack of published information about health-sector recovery. This paper draws lessons from a health system strengthening intervention conducted at district level during the recovery phase of the 2015 Nepal earthquake.

## Local setting

On 25 April 2015, an earthquake struck Nepal, followed by hundreds of aftershocks. Thirty-one of the country’s 75 districts were affected, out of which 14 highly affected ones were prioritized for rescue, relief and recovery operations.^8^ A total of 8897 deaths, including 18 among health workers and 22 310 people injured have been reported,^9^ while 662 (83.5%) of the 793 public health facilities in the 14 most affected districts were destroyed or partially damaged.^8^ More than 36 national and 137 international medical teams from 36 countries responded to the government of Nepal’s appeal for humanitarian response. About 117 000 patients were treated in outpatient departments, 41 200 were hospitalized and a total of 7124 surgical operations including 41 amputations were performed within 2 weeks.

**Fig. 1:**
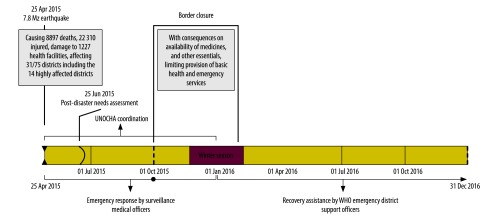
Timeline of events and World Health Organization support to district health authorities after the April 2015 Nepal earthquake

UNOCHA: United Nations Office for the Coordination of Humanitarian Affairs; WHO: World Health Organization.

An inter-agency cluster-based response was activated by the Government of Nepal. The health cluster^10^ was led by the Nepal Ministry of Health and co-led by the World Health Organization (WHO). For the immediate emergency response, the WHO facilitated the short-term deployment of Nepalese WHO staff working outside of Nepal on request from the health minister. They only remained in the country for the first few weeks after the disaster. In addition, from day 3 after the disaster, 14 WHO surveillance medical officers from the polio eradication and immunization preventable diseases programme of the WHO Nepal office were deployed in the 14 most-affected districts to assist the district health authorities in coordinating the health sector emergency response. However, from October 2015, with the relief period ending, surveillance medical officers had to return to their primary priorities of supporting the immunization programme (Fig. 1).

In December 2015, the people most affected by the earthquake were still living in temporary shelters, remaining vulnerable to disease and cold weather. The situation was made worse by a 4-month closure of the border between India and Nepal, resulting in shortages of fuel and medicines.^11^ The provision of health services was limited by the border closure and had not been re-assessed since the post-disaster needs assessment in May 2015. Out of 14 earthquake-affected districts, only five were regularly reporting disease surveillance data to the health ministry. Meanwhile, the United Nations Office for the Coordination of Humanitarian Affairs was phasing out support as the relief period came to an end, thus creating gaps in both coordination and monitoring during the recovery (Fig. 1).

## Approach

In January 2016, to address a perceived gap in support to the district health authorities in the earthquake-affected districts WHO posted 12 Nepalese medical doctors to the 14 most affected districts. There was one doctor to cover the well-served three central districts in Kathmandu valley and one each for the remaining 11 peripheral districts. These doctors, called WHO emergency district support officers, were to provide technical assistance to the district health authorities during the recovery phase.

The officers were recruited from within Nepal and trained following the regular procedures for recruitment and induction of WHO surveillance medical officers in Nepal. They all had some previous experience in humanitarian response. After a 1-week training on disaster and outbreak management and cluster coordination, they worked mainly from the offices of the district health authorities, but also frequently travelled to remote areas within their district. Guided directly for 6 months by a WHO international consultant, and later by senior WHO national staff, they were paid at national consultant rates similar to junior WHO surveillance medical officers in Nepal.

The role of the officers was first to assist the district health authorities in coordinating the organizations engaged in health-sector recovery activities. In their assigned districts, the officers helped organize meetings of the health cluster, mobilized resources from health-cluster partners when needed and followed progress in the reconstruction of health facilities.

Second, to strengthen disease surveillance and response, the officers facilitated the reporting of disease cases to the regular disease surveillance systems operated by the health ministry as well as the ad hoc post-earthquake disease surveillance. They also facilitated event-based surveillance and assisted the district health authorities and the district rapid response teams in investigating and containing outbreaks.

Third, the officers made field visits and monitored the recovery of health services and the reconstruction process of damaged health facilities using a standardized checklist. They also monitored the stocks of 20 essential medicines supplied by the health ministry. All operational challenges identified during the field visits to health facilities were discussed with the district health authorities to help in finding solutions.

The programme was gradually phased out from January 2017. In December 2017, only two officers were still providing part-time assistance to the district health authorities of the 14 most-affected districts. This was mainly to provide follow-up on reconstruction of health facilities.

Deployment of the emergency support officers (excluding travel and communication expenses) amounted to United States dollars 1500 per officer per month. We estimated that the cost of deploying these officers was about four times less than that of the average cost (salary and per diem) of deploying WHO international staff during the immediate response phase.

## Relevant changes

During the recovery period we observed several positive changes in the health sector situation in the affected districts (Table 1). The focus of coordination gradually shifted from emergency response to recovery activities. By December 2016, 375 health facilities (56.6% of all 662 damaged health facilities) had been repaired or rebuilt with support from various organizations. However, in 61 (20.1%) of 304 health facilities monitored after February 2016, support officers reported that buildings had been declared as unsafe by the authorities, with no major improvement over time. From mid-2016, health facilities were gradually able to provide the same level of services as in unaffected areas,^13^ including paediatric and adolescent services, follow-up of tuberculosis patients, management of respiratory infections and performing first aid.

**Table 1:** The outcomes of placing emergency district support officers in districts most affected by the April 2015 Nepal earthquake

Task	Situation pre-intervention (December 2015)	Inputs from emergency district support officers^a^	Situation post-intervention (December 2016)
Coordinating recovery activities in the health sector	District health authorities faced many competing health-sector priorities, e.g. preparing health-facility reconstruction plans, solving issues related to the border closure. The UN Office for the Coordination of Humanitarian Affairs was withdrawing.	Assisted district health authorities to prepare and conduct meetings of the health cluster. Completed the 4Ws matrix (Who is doing What, Where and When). Monitored recovery activities of health-sector partners (nongovernmental agencies, UN agencies working in health).	Health-cluster partners were mapped using the 4Ws approach. Partners were mobilized during responses to disease outbreaks and local disasters. Duplication of reconstruction plans and activities were avoided.
Strengthening disease surveillance and response	Few districts (5/14) were regularly reporting data to the Epidemiology and Control Division of the health ministry. Mean timeliness and completeness scores of districts were 41.7% and 66.7% respectively.	Coached medical reporters and statistic recorders. Assisted them in preparing statistical reports. Helped them solve logistic issues, e.g. with computers, electricity supplies or internet access.	Almost all health districts (13/14) were able to report data to the Epidemiology and Control Division.
	Rapid response teams were in place in all districts, with various levels of training, but without full capacity for outbreak investigations.	Participated in event-based surveillance and verification of rumours. Recorded 44 clusters of diseases over the year 2016. Technically and logistically assisted and trained rapid response teams during 33 outbreak investigations over the year 2016. Coordinated efforts with surveillance medical officers for the surveillance of vaccine-preventable diseases.	44 clusters of diseases affecting > 947 individuals were reported to district health authorities, investigated and contained. Rapid response teams were empowered. Information related to these outbreaks was shared with the Epidemiology and Control Division.
Monitoring the recovery of health facilities	Affected areas had 793 health facilities (723 health posts, 44 primary health-care centres, and 26 hospitals).^12^ The earthquake damaged 662 (83.5%) health facilities, including 363 (45.8%) destroyed.^12^ Due to logistic constraints, field supervision by district health authorities was rarely done, except for immunization activities. Availability and quality of health services was not known after the initial post-disaster needs assessment (May–June 2015). Most health facilities (36/41, 87.8%) were reporting medicine stock-outs.	Conducted 822 field visits in 451 different health facilities, using a standardized checklist developed from national health-facility surveys. Median number of visits per month to health facilities was 55.5 (interquartile range: 43.0–92.5). Of 302 health facilities visited from March–December 2016, 61 (20.1%) were using buildings declared unsafe by the health ministry. Looked for local solutions to decrease the risks for health teams and patients, e.g. the use of tents or relocation to public buildings. Provided real-time and accurate feedback to district health authorities regarding availability and quality of health-service provision, and daily challenges faced by health teams. Worked to improve inventory management of medicines and communication between health facilities.	Field visits showed that 375 health facilities were renovated or rebuilt (56.6% of all 662 damaged health facilities; 6 district hospitals, 10 primary health-care centres, 359 health posts). Availability of services was recorded and had recovered to pre-disaster levels. Most health posts monitored from June 2016 (75/85; 89.7%) had paediatric services and could manage respiratory tract infections; 75/87 (86.2%) had adolescent services available. and could follow-up tuberculosis patients; and 77/85 (88.5%) could perform first aid. However, sexually transmitted infections management and human immunodeficiency virus disease follow-up were available in only 31/87 (35.6%) and 7/87 (8.1%) health posts, respectively. Numbers of health facilities reporting medicine stock-outs decreased over the year from 36/41 (87.8%) in January 2016 to 48/58 (82.8%) in December 2016.

UN: United Nations.

^a^ Emergency district support officers were 12 Nepalese medical doctors posted by the World Health Organization (WHO) to the 14 districts most affected by the earthquake. They provided technical assistance to the district health authorities during the recovery phase.

Notes: Pre-intervention data mainly came from the post-disaster needs assessment conducted in May–June 2015 and led by the National Planning Commission with the assistance of national experts and institutions, Nepal’s neighbouring countries and development partners (UN agencies, international and national nongovernment agencies). Post-intervention data were collected by the WHO emergency district support officers during their monitoring visits to health facilities.

The proportion of health facilities reporting medicine stock-outs decreased over 2016 from 87.8% (36/41) in January 2016 to 82.8% (48/58) in December 2016, although shortages reappeared during the winter of 2016. The support officers identified several issues related to management of medicine stocks and resolved these with support from district health authorities. For instance, the officers facilitated the redistribution of medicines between health facilities according to need.

Reporting of disease surveillance data by districts greatly improved so that 13 out of 14 districts were able to report data to the health ministry. Timeliness and completeness gradually improved. The support officers gave technical and logistic support and training to district rapid response teams in the investigation and containment of 33 of the 44 disease outbreaks recorded over January to December 2016.

The support officers ensured there was regular mapping of health sector partners using a 4Ws matrix (Who is doing, What, Where and When). This facilitated early mobilization of partners during response to disease outbreaks or local emergencies such as landslides or flooding.

## Lessons learnt

Monitoring the delivery of health services was a realistic way to identify the challenges faced by health facilities and staff during the recovery phase and report these to the district health authorities. Many operational and contextual issues could then be quickly solved by the district health authorities. Even so, due to financial or programmatic constraints, issues such as the use of unsafe buildings remained unaddressed by December 2016 (Box 1).

Box 1 **Summary of main lessons learnt**

- Local medical doctors with some relevant experience, and closely guided by experienced WHO staff, can assist district health authorities in coordinating disaster recovery efforts and managing public health challenges during the recovery phase.
- Verifying rumours of disease outbreaks with field-level evidence, and early detection and containment of outbreaks, avoided escalation of incidents and allowed district health authorities to focus on recovery and reconstruction priorities.
- WHO emergency district support officers had no operational budget, and managing the expectations of district health authorities required explanations about the officers’ role in technical assistance and coordination support.

WHO: World Health Organization.

Explaining the role of the emergency support officers to the district health authorities was sometimes challenging. The officers had no operational budget to repair damaged health facilities or equipment or for medicines stock replenishment. Nor were they able to address issues directly. They identified problems, generated options, offered technical advice, facilitated decisions and assisted with data and information management and outbreak rapid response. Their main role was coordination between district health authorities, health-care providers and other health partners. Managing the expectations of the district health authorities regarding the work of the support officers required repeated explanations about their terms of reference and exact roles.

Verifying rumours of disease outbreaks with field level evidence was found to be crucial. This required the support officers to coordinate with the WHO surveillance medical officers, who remained in charge of supporting the district rapid response teams in surveillance and response of immunization preventable diseases. Early detection and containment of outbreaks, coupled with a rapid response to local emergencies, both of which are expected in post-disaster situations, avoided escalation of incidents and allowed district health authorities to focus on recovery and reconstruction priorities.

Well-performing emergency support officers were those with strong interpersonal skills, ability to learn and adapt quickly, and some background in public health. To avoid creating dependence of district health authorities and health partners on support officers, they were rotated from one district to another every 3 months and were gradually assigned to more than one district. We also observed that not all districts required the same level of support.

The cost‒effectiveness of this recovery assistance intervention could not be assessed using the data available, since it was not included in the programme design. If similar interventions had to be replicated in other settings, we would recommend assessing their cost‒effectiveness.
